# Metastatic Cervical Cancer in the Asia-Pacific Region: Current Treatment Landscape and Barriers

**DOI:** 10.1158/2767-9764.CRC-24-0647

**Published:** 2025-08-26

**Authors:** Jeffrey Chee-Hong Goh, Chyong-Huey Lai, Efren Javier Domingo, Jae Hoon Kim, Carmel Spiteri, Danny Hsu, Soo Yeon Ihm, Peng Peng

**Affiliations:** 1 Department of Medical Oncology, Royal Brisbane and Women's Hospital, Herston, Australia.; 2 Department of Obstetrics and Gynecology, Chang Gung Memorial Hospital, Linkou branch, Taoyuan, Taiwan.; 3 Department of Obstetrics and Gynecology, University of the Philippines College of Medicine-Philippine General Hospital, Manila, the Philippines.; 4 Department of Obstetrics and Gynecology, Gangnam Severance Hospital, Seoul, South Korea.; 5 MSD Australia, Macquarie Park, Australia.; 6 MSD Asia Ltd, Hong Kong, Hong Kong SAR, China.; 7 MSD Korea Ltd, Seoul, South Korea.; 8 Department of Obstetrics and Gynecology, Peking Union Medical College Hospital, Beijing, China.

## Abstract

Despite treatment advances for metastatic cervical cancer (mCC), the Asia-Pacific region faces significant barriers in treatment accessibility, availability, and healthcare infrastructure. This study explored the treatment landscape and barriers for mCC in the Asia-Pacific. A descriptive, cross-sectional, web-based study evaluating cervical cancer treatment patterns was conducted among medical, radiation, and gynecologic oncologists and gynecologists in the Chinese mainland (*n* = 80), Australia, the Philippines, South Korea, and Taiwan (*n* = 20 each). Eligible respondents were primarily involved in direct patient care (≥60%) and were key treatment deciders with ≥5 years of experience. Among patients with cervical cancer of 160 physicians, 10.9% had metastatic disease, of which 50.3% were aged 41 to 60 years and had Eastern Cooperative Oncology Group scores of 0 to 2 (78.7%). Top treatment modalities included systemic therapy (ST) alone (43.6%) and radiotherapy + ST (33.4%). Top first-line regimens were carboplatin/cisplatin + paclitaxel ± bevacizumab (42.3% and 33.1%, respectively), and the top second-line treatment regimens were carboplatin + paclitaxel + bevacizumab (12.0%) and cisplatin + paclitaxel + bevacizumab (11.5%). PD-L1 testing was more common in South Korea (80.8%) than in the Chinese mainland (48.8%) and Taiwan (26.4%). Treatment drivers included National Comprehensive Cancer Network guidelines (82.7%), disease stage (87.4%), Eastern Cooperative Oncology Group status (83.5%), comorbidities (59.1%), drug efficacy (88.2%), safety (84.3%), and accessibility (66.9%). Treatment challenges included poor prognosis (26.8%), patient affordability (21.3%), and limited treatment option availability (19.7%). In bevacizumab-reimbursed locations, patient tolerability and insufficient medical resources persisted. In conclusion, approximately 11% of cervical cancer cases were metastatic. Treatment preferences were radiotherapy and ST, with funding, cost, accessibility, and availability challenges. Policies supporting reimbursement and accessibility could encourage the adoption of effective alternative therapies.

**Significance:**

The findings offer valuable insights about current treatments and the related unmet needs in funding, cost, accessibility, and availability across the Asia-Pacific region. These further highlight areas of importance and the need for implementing reimbursement policies and enhancing accessibility to support the adoption of effective, advanced treatments.

## Introduction

As the fourth most common cancer in women, the 5-year survival rate for cervical cancer in developed locations is greater than 70%, whereas in low-income regions, it is less than 60% (1). In 2020, Asia (Eastern, Southeastern, South-central, and Western) accounted for 58% of global cervical cancer cases (1). In Australia, the incidence of cervical cancer between 2011 and 2015 was reported at 6.3 per 100,000, with a mortality rate of 1.4 per 100,000 between 2014 and 2018 (2). Although seemingly low by global standards, substantial inequities persist due to limited accessibility to treatment centers in rural areas, with incidence rates higher among Indigenous women compared with non-Indigenous women (2). The Chinese mainland accounts for a third of the global burden of cervical cancer, with 150,700 new cases and 55,700 deaths reported in 2022 (3). As the third most common cancer in women, South Korea reported an incidence rate of 18.9 per 100,000 in 2019 (4). In Taiwan, cervical cancer was ranked as the eighth cause of mortality among women (5), and in the Philippines, as the second most common cancer and a leading cause of cancer mortality among women, cervical cancer accounts for 14% of cases (6).

Women diagnosed with metastatic cervical cancer (mCC; stage IVb) have a poor prognosis, with a 17.6% 5-year survival rate, and most occurrences are attributed to failures in early detection (7). The limited information on estimates of mCC cases could potentially be attributed to various detection methods affected by a location’s socioeconomic status. Furthermore, limited awareness and accessibility issues, especially in rural and geographically challenged areas, may have led to underreporting of cases (8–10).

Based on  the International Federation of Gynecology and Obstetrics (FIGO) 2018 classification, for patients with stage IVb cervical cancer, the American National Comprehensive Cancer Network (NCCN) guidelines recommend individualized external beam radiotherapy, along with systemic therapy (ST; ref. 11). In recent years, there have been notable treatment advances for mCC. Bevacizumab, an angiogenesis inhibitor, in combination with standard-of-care chemotherapy (carboplatin or cisplatin + paclitaxel), is widely approved as a first-line (1L) therapy. Pembrolizumab, an immune checkpoint inhibitor (ICI), in combination with chemotherapy, with or without bevacizumab, has been approved as a 1L treatment for patients with mCC with a combined positive score ≥1 by the FDA in 2021 (12) and by the Therapeutic Goods Administration for Australia, the Ministry of Food and Drug Safety for South Korea, and the FDA in Taiwan and the Philippines in 2022 (13–16). Findings from the KEYNOTE-826 trial demonstrated that pembrolizumab, in combination with platinum-based chemotherapy regimens such as paclitaxel + cisplatin or carboplatin ± bevacizumab, improved progression-free survival (PFS) and overall survival (OS) in the 1L setting. These results have been incorporated into several clinical guidelines, including those in the Chinese mainland (17).

Despite treatment advances, limitations about healthcare infrastructure, treatment accessibility, and availability persist in the Asia-Pacific region (18, 19). As the fastest-growing economic region with a diverse group of income levels (18), long-term conditions such as cervical cancer become expensive to manage. A combination of insufficient public coverage, lack of reimbursement, and the heavy disease burden exposes patients to high out-of-pocket treatment costs, especially for innovative therapies, leading to suboptimal treatment outcomes (8, 9).

To encourage governmental organizations and policymakers to address these issues and assess the “fit” to their respective locations for maximum impact and sustainability, it is imperative to understand the current real-world treatment patterns for mCC and its associated unmet patient needs. In locations within the Asia-Pacific, such as Australia, the Chinese mainland, the Philippines, South Korea, and Taiwan, there remains a disparity in information about the overall treatment-associated resource utilization and the available treatments and regimens used by patients with mCC. Hence, the study was conducted to explore the patient characteristics and the current treatment landscape across the five locations. More importantly, it aimed to identify unmet needs in existing treatment regimens, such as challenges with accessibility and healthcare resources faced by patients with mCC in the region.

## Materials and Methods

### Data sources

This was a descriptive, cross-sectional, moderator-assisted, web-based study across five locations (Australia, the Chinese mainland, the Philippines, South Korea, and Taiwan) involving physician interviews comprising both quantitative and qualitative sections. This study and the publication of subsequent results were approved by the local Institutional Review Board of each location (approval number: Australia, HREC2022-03-255; the Chinese mainland, SECCR/2022-131-01 and SECCR/2022-131-02; the Philippines, UPMREB 2022-0457-EX; South Korea, P01-202305-01-040; and Taiwan, 202201280B0A3 and 202201280A3C601).

### Study design and population

Medical, radiation, and gynecologic oncologists and gynecologists were interviewed in each location for 60 minutes (Australia: *n* = 20, the Chinese mainland: *n* = 80, the Philippines: *n* = 20, and Taiwan: *n* = 20) and for 40 minutes in South Korea due to constraints on the length of the interview (*n* = 20; Supplementary Fig. S1). The distribution of specialists was designed to ensure a representative sample of care in each location. Screening was conducted via phone calls across all locations, and those meeting the inclusion criteria were recruited into the study, following which interviews were conducted either by phone or through virtual meeting platforms, such as Zoom, Microsoft Teams, and VooV Meeting. Physicians were asked to respond based on their clinical experiences over the past 6 months.

Purposive sampling was carried out to recruit eligible cervical cancer specialists who were board-certified, provided consent, had ≥5 years of experience, were main treatment decision-makers, treated ≥30 patients with cervical cancer in the past 6 months (except in South Korea: treated 20 patients with cervical cancer), and had spent ≥60% of their time in direct patient care. These criteria, discussed with expert clinicians, ensured that physicians were specialized enough in the disease to respond to questions related to cervical cancer management and share their opinions. There were no exclusion criteria. Respondents recruited were from public, private, or university healthcare settings.

### Statistical analysis

Quantitative data collected from close-ended questions were analyzed descriptively using counts, percentages, means, and SDs, depending on the scale (nominal, ordinal, or continuous) of the variable. Qualitative open-ended data from the interview transcripts were analyzed using NVivo (v1.7.1; NVivo, RRID:SCR_014802) qualitative data analysis software (20).

For qualitative insights, prior studies suggest that data saturation is typically achieved with at least 12 respondents (21). A sample of 20 respondents per location (excluding the Chinese mainland) was sufficient for qualitative insights, whereas a sample of 80 for the Chinese mainland ensured geographic representation. No power calculations were necessary, as quantitative insights were exploratory in nature.

### Data availability

All data files and data management/analysis syntax are stored on Oracle Life Sciences cloud-based servers, to which permission to access files is only granted to appropriate team members. Data will be made available from the corresponding author upon request.

## Results

### Physician-reported patient characteristics

**Figure 1:**
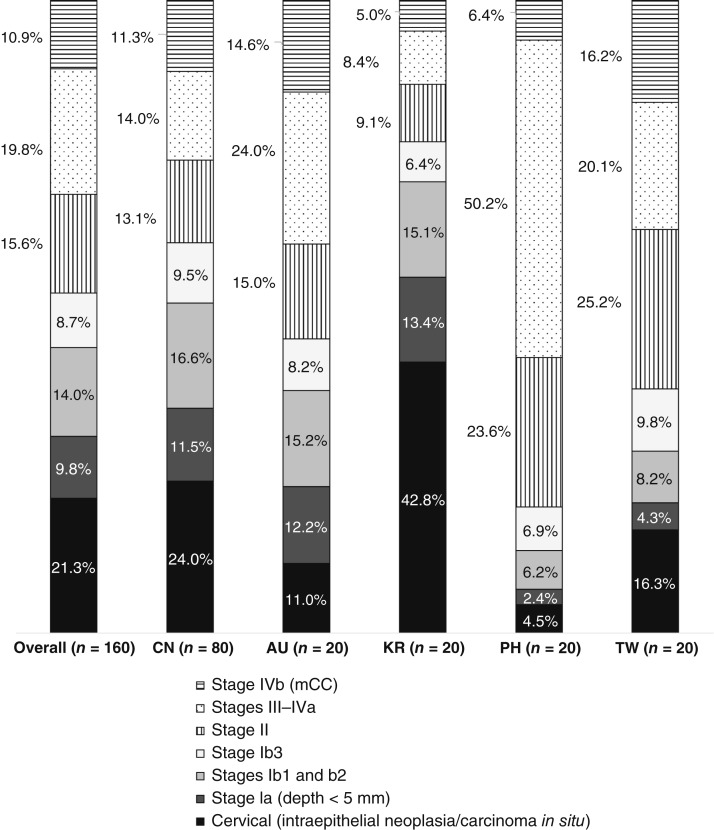
Patient caseload based on staging by FIGO 2018. AU, Australia; CN, Chinese mainland; PH, Philippines; TW, Taiwan.

According to the 160 recruited physicians, approximately 1 in 10 of their patients with cervical cancer had metastatic disease (10.9%). The highest patient caseload was reported in Taiwan (16.2%), and the lowest was reported by physicians in South Korea (5.0%; Fig. 1).

About half of the patients with mCC reported by the physicians (50.3%) were aged between 41 and 60 years, with the highest proportion observed in the Philippines (68.2%). Physicians from South Korea and Taiwan reported that 59.6% and 58.8% of patients with mCC, respectively, were aged >60 years, with Taiwan additionally reporting the lowest incidence among patients aged <30 years (0.6%). Physicians reported that their patients had Eastern Cooperative Oncology Group (ECOG) scores of 0 to 2 (78.7%), hypertension (30.5%), and diabetes (24.5%) and more than half (50.7%) were not living within close proximity to treatment centers. The majority had no treatment choice preference (74.1%), except for 45.0% of patients in the Philippines who had treatment preferences (Table 1).

**Table 1:** Mapping of characteristics of patients with mCC based on physicians’ responses

​	Region (%)	By location				
		CN (%)	AU (%)	KR (%)	PH (%)	TW (%)
Age distribution, years						
≤30	1.7	2.0	1.6	1.4	2.7	0.6
31–40	8.2	8.3	13.2	3.0	13.5	4.1
41–50	19.2	17.3	23.8	11.0	37.9	12.1
51–60	31.1	36.4	28.5	25.1	30.3	24.3
61–70	23.7	21.8	16.0	37.0	12.1	32.6
>70	16.1	14.2	16.8	22.6	3.5	26.2
ECOG performance status						
0	27.7	20.3	33.2	39.7	23.2	35.8
1	29.7	31.4	38.8	28.3	17.2	29.2
2	21.3	20.3	18.8	17.9	30.0	22.5
3	15.2	20.8	7.5	10.7	18.2	7.8
4	6.1	7.3	1.6	3.5	11.3	4.7
Comorbidities						
Hypertension	30.5	27.8	30.0	31.5	39.1	30.0
Diabetes	24.5	18.0	27.9	28.3	37.7	24.6
Renal disease	15.6	9.0	18.2	16.2	36.5	12.7
Heart disease	13.8	11.3	18.5	12.3	13.8	18.5
Gastrointestinal disease	10.3	11.7	5.9	13.3	8.5	8.3
Cerebrovascular disease	9.1	9.1	7.9	10.2	8.6	9.7
Liver disease	8.2	8.4	4.4	7.9	10.6	8.9
Musculoskeletal disease	8.0	6.2	7.9	11.0	4.2	13.5
Anemia	0.8	0.8	0.0	0.0	3.5	0.0
High cholesterol/hyperlipidemia	0.6	0.0	0.0	3.8	0.0	0.0
Lung disease	0.3	0.3	0.0	1.0	0.0	0.0
Pneumonia	0.3	0.6	0.0	0.0	0.0	0.0
Psychiatric/psychologic symptoms (depression and anxiety)	0.2	0.0	0.0	0.0	1.8	0.0
Insomnia	0.2	0.0	0.0	0.0	1.8	0.0
Respiratory diseases (asthma, tuberculosis)	0.2	0.1	1.2	0.0	0.0	0.0
Hematologic system disease	0.2	0.4	0.0	0.0	0.0	0.0
Cancer metastasis	0.2	0.4	0.0	0.0	0.0	0.0
Autoimmunity diseases	0.1	0.1	0.0	0.0	0.0	0.6
Genital tract inflammation	0.1	0.3	0.0	0.0	0.0	0.0
Pulmonary fibrosis	0.1	0.2	0.0	0.0	0.0	0.0
Chronic bronchitis	0.0	0.1	0.0	0.0	0.0	0.0
Emphysema	0.0	0.1	0.0	0.0	0.0	0.0
Systemic lupus erythematosus	0.0	0.1	0.0	0.0	0.0	0.0
Hepatitis C	0.0	0.0	0.0	0.0	0.0	0.0
Dementia	0.0	0.0	0.0	0.1	0.0	0.0
Not applicable/none	0.4	0.4	1.5	0.0	0.0	0.0
Proximity to treatment center						
Patients living in close proximity to treatment center (e.g., for radiotherapy; traveling time: 30–59 minutes)	49.3	39.5	70.9	47.0	60.0	51.5
Patients not living in close proximity to treatment center (e.g., for radiotherapy; traveling time: ≥60 minutes)	50.7	60.6	29.1	53.0	40.0	48.5
Patient preference						
Patients have their own preference on drug options and are heavily involved in making joint decisions on treatment choice along with the attending physician	25.9	23.1	35.9	8.7	45.0	26.4
Patients have no preference on drug options and rely on the attending physicians for recommendations	74.1	76.9	64.1	91.4	55.0	73.6

Abbreviations: AU, Australia; CN, Chinese mainland; KR, South Korea; PH, Philippines; TW, Taiwan.

### Current treatment modalities and regimens

Overall, top treatment modalities included ST alone (43.6%) and radiotherapy + ST (including concurrent chemoradiotherapy; 33.4%; Supplementary Table S1). For 1L treatments, carboplatin + paclitaxel ± bevacizumab (42.3%) and cisplatin + paclitaxel ± bevacizumab (33.1%) combinations were the top regimens (Table 2). The highest proportion of patients receiving carboplatin + paclitaxel + bevacizumab (47.8%) was reported by the physicians for Australia and cisplatin + paclitaxel + bevacizumab (48.5%) for South Korea. All locations reported the use of bevacizumab + chemotherapy combinations, with the Philippines reporting the lowest use at 7.4%. Patients in South Korea received immunotherapy combinations (1.5%) as 1L treatments (Table 2).

**Table 2:** First-line treatment regimens received by patients with mCC

​	Region (%)	By location				
		CN (%)	AU (%)	KR (%)	PH (%)	TW (%)
Carboplatin + paclitaxel	23.5	18.7	16.6	14.5	61.1	18.6
Bevacizumab + cisplatin + paclitaxel	21.2	20.2	10.6	48.5	1.2	21.8
Bevacizumab + carboplatin + paclitaxel	18.8	22.0	47.8	0.8	6.1	15.1
Cisplatin + paclitaxel	11.9	12.4	3.3	11.3	12.1	18.3
Cisplatin	6.5	1.5	11.4	9.0	7.9	13.1
Cisplatin + docetaxel	2.2	3.5	2.5	0.0	0.9	1.7
Carboplatin + docetaxel	2.1	3.9	1.9	0.0	0.9	0.3
Investigational drug	2.1	2.4	2.2	4.5	0.0	0.3
Bevacizumab + topotecan + paclitaxel	2.1	3.1	0.0	4.5	0.1	0.1
Bevacizumab + topotecan + cisplatin + paclitaxel	1.9	4.3	0.0	0.0	0.0	0.0
Cisplatin + gemcitabine	1.0	1.1	1.6	0.0	1.2	1.4
Nedaplatin + paclitaxel	1.0	2.3	0.0	0.0	0.0	0.0
Carboplatin	1.0	0.2	2.2	2.5	1.5	0.6
Cisplatin + radiotherapy	0.8	0.0	0.0	0.0	5.9	0.0
Cisplatin + radiotherapy after bevacizumab + cisplatin + paclitaxel	0.6	0.4	0.0	3.0	0.0	0.0
Bevacizumab + cisplatin + paclitaxel + pembrolizumab	0.5	0.5	0.0	1.5	0.0	0.0
Nedaplatin + irinotecan	0.4	1.0	0.0	0.0	0.0	0.0
Topotecan	0.4	0.0	0.0	0.0	0.0	2.8
Cisplatin + vincristine	0.4	0.0	0.0	0.0	0.0	2.8
Topotecan + cisplatin	0.3	0.0	0.0	0.0	0.0	2.2
Anti–PD-1 + chemotherapy	0.2	0.6	0.0	0.0	0.0	0.0
Anti–PD-1 + cisplatin + paclitaxel	0.2	0.5	0.0	0.0	0.0	0.0
Docetaxel + bevacizumab + cisplatin	0.2	0.5	0.0	0.0	0.0	0.0
Bevacizumab	0.2	0.0	0.0	0.0	1.2	0.0
Targeted therapy	0.1	0.3	0.0	0.0	0.0	0.0
Pembrolizumab	0.1	0.0	0.0	0.0	0.0	0.6
Nivolumab	0.1	0.0	0.0	0.0	0.0	0.6
Camrelizumab + carboplatin + paclitaxel	0.1	0.2	0.0	0.0	0.0	0.0
Paclitaxel + gemcitabine + platinum	0.1	0.2	0.0	0.0	0.0	0.0
Lobaplatin	0.0	0.1	0.0	0.0	0.0	0.0
TCM	0.0	0.0	0.0	0.0	0.0	0.0
Paclitaxel + nedaplatin	0.0	0.0	0.0	0.0	0.0	0.0
Not applicable	0.0	0.1	0.0	0.0	0.0	0.0

Abbreviations: AU, Australia; CN, Chinese mainland; KR, South Korea; PH, Philippines; TCM, traditional Chinese medicine; TW, Taiwan.

**Table 3:** Second-line treatment regimens received by patients with mCC

​	Region (%)	By location				
		CN (%)	AU (%)	KR (%)	PH (%)	TW (%)
Carboplatin + paclitaxel + bevacizumab	12.0	11.9	9.1	4.5	15.9	19.8
Cisplatin + paclitaxel + bevacizumab	11.5	11.9	4.1	5.5	6.5	28.3
Cisplatin + gemcitabine	7.5	6.8	11.2	5.8	9.7	6.1
Investigational drug	6.3	3.8	29.7	0.0	2.9	2.2
Carboplatin + paclitaxel	5.0	4.3	6.5	0.5	12.4	3.6
Bevacizumab	4.8	4.0	14.4	0.0	6.8	1.4
Topotecan + cisplatin	4.5	0.0	0.0	26.0	2.1	0.6
Topotecan	4.2	0.7	5.0	5.3	5.9	11.3
Bevacizumab + topotecan + paclitaxel	3.8	4.7	5.9	1.5	0.0	5.4
Cisplatin + paclitaxel	3.2	3.8	0.9	2.5	1.2	6.4
Pembrolizumab	2.8	2.4	0.0	8.0	0.3	3.6
Cisplatin + docetaxel	2.7	6.0	0.0	0.3	0.6	0.3
Carboplatin + docetaxel	2.7	1.9	0.0	6.5	5.9	0.3
Cisplatin	2.2	3.7	0.6	1.5	0.6	1.1
Docetaxel	2.0	1.6	0.3	1.3	7.9	0.2
Carboplatin	1.9	2.2	5.3	0.0	1.5	0.3
Bevacizumab + topotecan + cisplatin + paclitaxel	1.8	4.1	0.0	0.0	0.0	0.0
Gemcitabine only	1.5	0.0	0.0	0.0	11.2	0.0
Cisplatin + 5-FU	1.5	0.0	0.0	9.5	0.0	0.0
Nedaplatin + paclitaxel	1.4	3.2	0.0	0.0	0.0	0.0
Cisplatin + etoposide	1.1	1.9	0.0	2.0	0.0	0.0
Cisplatin + vinorelbine	1.1	1.0	0.0	0.0	0.0	5.0
Cisplatin + topotecan	1.1	2.6	0.0	0.0	0.0	0.0
Nedaplatin + irinotecan	1.1	2.6	0.0	0.0	0.0	0.0
Irinotecan + S-1	1.1	2.5	0.0	0.0	0.0	0.0
Paclitaxel	1.1	1.2	1.2	1.5	0.9	0.3
Bevacizumab + cisplatin + paclitaxel + pembrolizumab	1.0	0.0	0.0	1.5	5.9	0.0
Irinotecan	0.9	0.7	0.0	3.5	0.0	0.6
Topotecan + paclitaxel	0.7	0.0	0.0	1.5	0.0	3.3
Gemcitabine	0.7	1.6	0.0	0.0	0.0	0.0
S-1 (tegafur, gimestat, otastat)	0.5	1.2	0.0	0.0	0.0	0.0
Cisplatin + RT after bevacizumab + cisplatin + paclitaxel	0.4	1.0	0.0	0.0	0.0	0.0
Gemcitabine + bevacizumab + cisplatin	0.3	0.7	0.0	0.0	0.0	0.0
Nedaplatin	0.3	0.7	0.0	0.0	0.0	0.0
Bevacizumab + cisplatin	0.3	0.0	0.0	0.0	2.1	0.0
Entrectinib	0.3	0.6	0.0	0.0	0.0	0.0
TCM	0.3	0.6	0.0	0.0	0.0	0.0
Pembrolizumab + chemo	0.2	0.5	0.0	0.0	0.0	0.0
PD-1 + platinum + gemcitabine	0.2	0.4	0.0	0.0	0.0	0.0
Vinorelbine	0.2	0.4	0.0	0.0	0.0	0.0
Targeted therapy	0.2	0.4	0.0	0.0	0.0	0.0
Cisplatin + entrectinib	0.2	0.0	0.0	1.0	0.0	0.0
Etoposide	0.1	0.2	0.0	0.0	0.0	0.0
Cisplatin + irinotecan	0.1	0.0	0.0	0.5	0.0	0.0
Lobaplatin	0.0	0.1	0.0	0.0	0.0	0.0
Lobaplatin + paclitaxel	0.0	0.1	0.0	0.0	0.0	0.0
PD-1 + paclitaxel + platinum	0.0	0.1	0.0	0.0	0.0	0.0
Not applicable	3.4	2.5	5.9	10.0	0.0	0.0

Abbreviations: 5-FU, 5-Fluorouracil; AU, Australia; CN, Chinese mainland; KR, South Korea; PH, Philippines; RT, radiotherapy; TCM, Traditional Chinese medicine; TW, Taiwan.

Carboplatin + paclitaxel + bevacizumab and cisplatin + paclitaxel + bevacizumab were the top second-line treatment regimens (12.0% and 11.5%, respectively), with the highest proportion of physicians reporting that their patients received the carboplatin (19.8%) and cisplatin (28.3%) combinations in Taiwan. In South Korea, physicians reported that their patients received immunotherapy (pembrolizumab monotherapy: 8.0%; cisplatin + paclitaxel + bevacizumab + pembrolizumab: 1.5%; Table 3).

### Biomarker testing

PD-L1 testing was the highest in South Korea (80.8%) and implemented to a lesser extent in the Chinese mainland (48.8%) and Taiwan (26.4%; Supplementary Fig. S2). Additionally, physicians in South Korea reported the highest likelihood for future PD-L1 (95.0%) and microsatellite instability/mismatch repair (50.0%) testing (Supplementary Fig. S3). Across the three locations, qualitative insights revealed that patient selection for biomarker testing depended on those who had good financial stability and were fitter (Supplementary Table S2).

### Factors influencing physicians’ treatment choices

**Figure 2:**
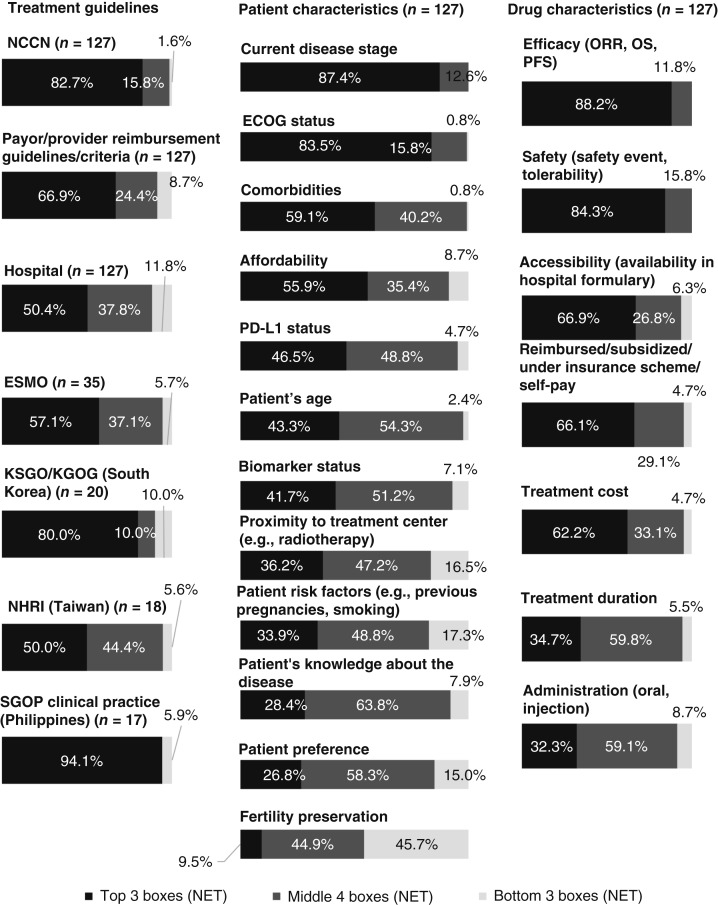
Regional responses on factors influencing physicians’ treatment choices for patients with mCC. The importance of each factor was rated on a scale of 1 (not important at all) to 10 (extremely important), with scores categorized into the top three boxes (10/9/8), middle four boxes (7/6/5/4), and bottom three boxes (3/2/1). ESMO, European Society of Medical Oncology; KSGO/KGOG, Korean Society of Gynecologic Oncology/Korean Gynecologic Oncology Group; NET, net total; NHRI, National Health Research Institute; ORR, overall response rate; SGOP, Society of Gynecologic Oncologists of the Philippines.

Overall, the top treatment guidelines affecting physicians’ treatment decisions included NCCN (82.7%), payor/provider reimbursement (66.9%), and European Society for Medical Oncology guidelines (57.1%; Fig. 2). Hospital guidelines were important in the Philippines (76.5%) and Taiwan (88.9%), and local guidelines were valued in the Philippines (94.1%) and South Korea (80.0%; Supplementary Table S3).

In terms of patient characteristics, current disease stage (87.4%), ECOG status (83.5%), and comorbidities (59.1%) were reported as important treatment choice drivers (Fig. 2). Physicians in the Philippines also reported high importance (88.2%) for both patient preference and proximity to treatment center, affordability (82.4%), and disease knowledge (70.6%; Supplementary Table S3).

Across all locations, drug efficacy (88.2%), safety (84.3%), and accessibility (66.9%) were the top treatment choice drivers (Fig. 2). Physicians in the Philippines, South Korea, and Taiwan reported treatment cost (94.1%, 60.0%, and 83.3%, respectively) and accessibility (82.4%, 75.0%, and 88.9%, respectively) as highly important. Additionally, insurance reimbursement was valued in South Korea (80.0%) and Taiwan (94.4%; Supplementary Table S3).

### Physician-reported unmet needs of patients based on treatment option availability

**Figure 3:**
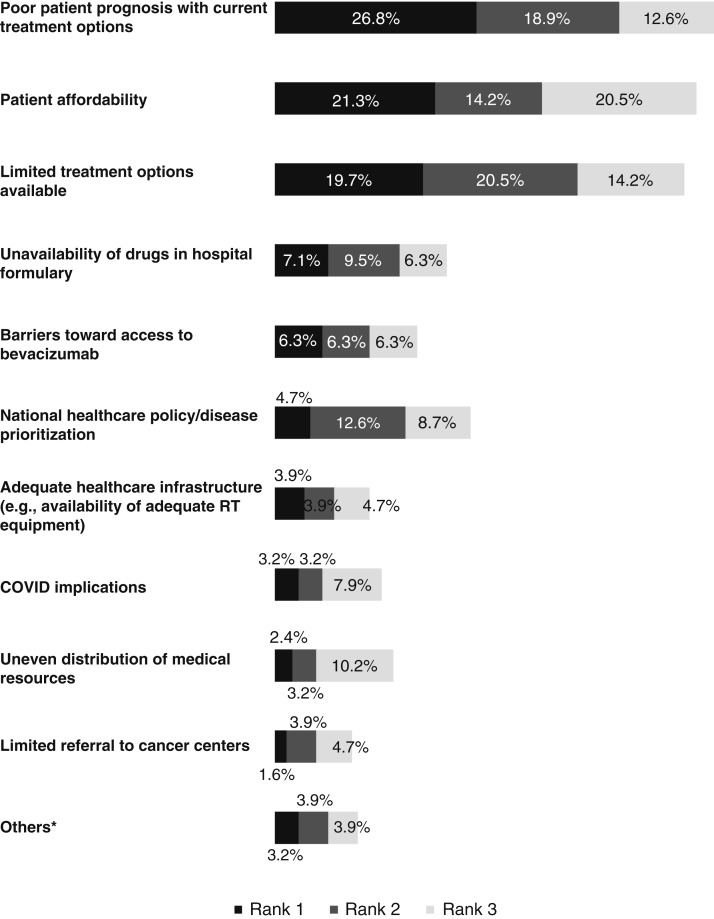
Physician-reported unmet needs affecting patients based on the treatment options available. Unmet needs were scored using a ranking system based on the level of impact: 1, biggest impact; 2, second biggest impact; 3, third biggest impact. *, Lack of skilled medical personnel, nutrition, opting for alternative medicine and herbal remedies, patients’ willingness to receive treatment, poor profitability of the hospital, immuno-oncology reimbursement not being applied/limitations in the reimbursement criteria, giving up treatment due to old age, side effects due to ST, and not applicable. COVID, coronavirus disease; RT, radiotherapy.

The top three unmet needs reported by physicians were poor patient prognosis with current treatment options (26.8%), patients’ ability to afford treatment (21.3%), and limited treatment options available (19.7%; Fig. 3). Poor patient prognosis from current treatment options was most impactful in Australia (29.4%), the Chinese mainland (29.1%), and Taiwan (27.8%), whereas patient affordability affected South Korea (30.0%) and the Philippines (52.9%) the most. Limited treatment option availability was also among the most impactful challenges reported for the Chinese mainland (21.8%), South Korea (25.0%), and Taiwan (22.2%; Supplementary Table S4).

Physicians’ perceptions of bevacizumab reimbursement varied across the region. The majority of physicians in Taiwan (88.9%) and Australia (76.5%) reported reimbursement for bevacizumab, whereas 74.6% in the Chinese mainland and 94.1% in the Philippines perceived that bevacizumab was not reimbursed (Supplementary Fig. S4). In locations without reimbursement, patient affordability was reported as the biggest barrier to bevacizumab accessibility (Supplementary Table S5). Despite available reimbursement, qualitative insights revealed that challenges such as patient tolerability (“*If they have bowel diseases like bowel involvement, we don’t give it; or they have comorbidities like a history of deep vein thrombosis, or high blood pressure.*”—CS00105, AU) and lack of medical resources (“*Basically, it goes around chemo beds for these patients. They only have a certain number of chairs/beds, they can only give treatment to such number of patients per day. If there’s a backlog, then patients cannot be fitted in on a timely matter. So, it’s about the resources of chemo department.*”—CS00106, AU; Supplementary Table S6) remained. The current reimbursement status for each location is as provided in Supplementary Table S7.

## Discussion

Overall, physicians reported that approximately 11% of their patients with cervical cancer had mCC, with the highest proportion reported in Australia and Taiwan and the lowest in South Korea. The current results showed differences between the locations. Despite the availability of free vaccination and screening programs, studies have reported women who lapsed in screening or had never been screened for human papillomavirus (22). The increased detection and availability of treatment facilities in urban areas, plus limited awareness and accessibility to treatment in rural populations, hinder early detection in some Indigenous populations and among those from rural areas (23, 24).

More than half of reported patients with mCC were aged 41 to 60 years, similar to studies across Asia (25). The higher incidence in patients aged >60 years reported by physicians in South Korea and Taiwan compared with other locations may be due to a decreased number being screened and the introduction of prevention programs after the 1990s. Furthermore, therapeutic dilemmas due to underrepresentation and increased comorbidities among the elderly have led to relatively conservative treatment patterns, resulting in a poorer prognosis (26). Contrastingly, the free prevention programs in both locations (27, 28) could have benefited the younger population, accounting for the lower rates reported in those aged <30 years. The current findings highlight the real-world effectiveness and importance of efforts from the governments and relevant funding organizations in prevention and early detection. Furthermore, the increasing medical costs for the elderly due to multimorbidities and higher disease severity suggest the need for revisiting funding policies for this age group as well.

Treatment modalities received by patients such as ST, followed by radiotherapy + ST, were in line with studies recommending advanced treatment and combinatory approaches with the inclusion of angiogenesis inhibitors and ICIs (29). The implementation of bevacizumab combinations as top 1L treatment choices corresponded to recommended NCCN guidelines (30). Increased use of carboplatin or cisplatin + paclitaxel + bevacizumab in locations such as Australia and South Korea may possibly be due to physicians having fewer concerns about feasibility for patients, as there is reimbursement in place and increased bevacizumab accessibility (31). The same combinations were received by the highest proportion of patients with mCC for second-line treatment. The addition of bevacizumab has shown improved OS in a phase III clinical trial when added to standard-of-care combination chemotherapeutic regimens (32). However, disparities in treatment costs for bevacizumab potentially contribute to restricted accessibility, especially in locations where patients incur the full cost of treatment or reimbursement is only partial (33). In Chinese mainland and South Korea, bevacizumab is reimbursed but typically requires a co-payment or medical insurance (34–36); it is fully reimbursed in Australia. In the Philippines, no reimbursement is available, whereas Taiwan remains the only location offering full reimbursement for bevacizumab in mCC (37, 38). Hence, affordability may remain a concern, particularly in low-to-middle–income countries and for those needing financial medical assistance. With appropriate approval and reimbursement in place, the bevacizumab + chemotherapy regimens potentially pave the way for combination therapy utilizing immunotherapy agents, such as pembrolizumab, authorized for use in patients with mCC in some locations (31, 39). Despite existing reimbursement policies for bevacizumab in some locations, some physicians were unsure or reported no reimbursement. This perception could stem from the lack of awareness among physicians about updated reimbursement policies, especially for advanced therapies. The lack of awareness may be due to limitations such as healthcare infrastructure, access to updated information, and continuous professional training. Furthermore, challenges in certain locations, such as a lack of medical personnel and increased patient load at certain facilities, may have contributed to physicians lacking sufficient time to update themselves on reimbursement policies on a regular basis (40). This predisposes patients to suboptimal outcomes, as affordability influences treatment decisions in addition to the geographic challenges that may already be hindering treatment accessibility, especially for those in the outer islands and rural areas (2, 24).

Overall, close to half of patients received PD-L1 biomarker testing, with the highest reported in South Korea and, to a lesser extent, in the Chinese mainland and Taiwan. In addition, physicians in the Chinese mainland and South Korea reported the highest likelihood for future biomarker testing. Although physicians in the Chinese mainland reported the highest likelihood for future PD-L1 testing, a lesser extent of patients having received the testing was observed. This may be attributed to the anticipation that future reimbursement policies may likely cover the costs of advanced treatments following the identification of positive test results, hence increasing the possibility of recommending it to patients. PD-L1 testing potentially identifies patients who may benefit from immunotherapy treatment based on treatment guidelines or approved indications. The KEYNOTE-826 trial reported significant increases in OS, PFS, response duration, and quality of life in recipients of pembrolizumab, platinum-based chemotherapy, and bevacizumab (32). However, despite usage approval in South Korea and Taiwan, only a small percentage of patients were given pembrolizumab combinations as 1L therapy. Physicians reported that low recommendations to patients were attributed to feasibility barriers as reimbursement remained a challenge. Hence, biomarker testing was also limited in certain locations and recommended based on patients’ ability to afford the suggested treatment, except in South Korea, where testing was prevalent due to the reimbursement policy in place (41). Furthermore, the decreased proportion of patients receiving advanced treatments in Taiwan may be attributed to the fact that ICIs covered by National Health Insurance are limited to several cancers, excluding cervical cancer, implicating a lack of advanced therapy management for cervical cancer (42). Although the Chinese mainland has yet to receive approval at the time of this study, South Korea equally faces challenges in immunotherapy reimbursement, with limited to no reimbursement for patients with cervical cancer undergoing treatment. Hence, these barriers could potentially underline the limited biomarker testing observed, further hindering optimal treatment for mCC. Additionally, based on the lower likelihood of future testing for biomarkers reported by physicians across Taiwan and varying healthcare equity, policies, and governmental subsidizations across locations, the findings further implicate potential challenges in affordability and the importance of reimbursement policies, especially for advanced therapies (19).

Physicians reported NCCN guidelines as the main treatment choice driver, coinciding with its implementation as the gold standard due to recognition, reliability, and recommended standardized treatments for patients with mCC (30). The high importance of reimbursement guidelines could be attributed to the increased cost of advanced treatment, hence serving as a reference source to assist patients in obtaining listed reimbursed treatments to overcome unaffordability (43), whereas the importance of local and hospital guidelines reported, which are based on NCCN, could be due to a better reflection of the territory’s clinical landscape and healthcare infrastructure (44).

Patient comorbidities equally affected treatment choices as conditions such as poorly controlled hypertension prohibited patients from receiving bevacizumab treatment. As bevacizumab functions by inhibiting VEGF, which predisposes individuals to hypertension development, an appropriate treatment course would be necessary (45). In the Philippines, proximity to treatment centers, disease knowledge, and treatment preference held higher importance. This was potentially due to medical ethics requiring full disclosure of treatment plans for patient consent (46); hence, disease knowledge was equally important. The lack of reimbursement for cervical cancer may account for the importance of closer proximity to treatment to decrease the financial burden on patients. This is supported by studies implicating factors such as government healthcare policies, medical infrastructure, and socioeconomic status that govern treatment choices and differ across locations (18). Moreover, drug accessibility and treatment costs were also important in locations where the potential geographic distribution of treatment centers and reimbursement challenges hindered patients from obtaining optimal treatment although drug safety and efficacy remained most important, possibly due to OS and PFS as the main determinants of treatment suitability (40).

Despite treatment advances globally, access to targeted therapies and immunotherapy in many parts of the Asia-Pacific remains limited. With the high costs of targeted therapies and their increasing use due to the expected increase in the disease burden over the next two decades (19), studies such as this help inform policy and program implementation to reduce inequities and assist patients with mCC in obtaining optimal treatment outcomes. Insufficient public coverage and reimbursement for innovative therapies, coupled with the heavy disease burden (1), lead to high out-of-pocket treatment costs (18). Although this only seems a challenge for low-to-middle–income locations, incidence and mortality rates among Indigenous, rural-dwelling, and poorer patients have also been observed in higher-income locations (10, 24). Additionally, lower rates of screening have been observed in these patient populations (10). This highlights the existence of challenges faced by patients regionally and the room for improvement. Ultimately, effective responses to increase funding for high-cost effective treatments require strengthening all functions within the healthcare system: resource generation, financing, provision of services, and leadership (19).

Despite the growth in clinical oncology trials, significant disparities in trial distribution and access remain, especially in low-to-middle–income countries across the Asia-Pacific region. Factors contributing to these challenges include variations in clinical trial infrastructure, economic constraints, and differing regulatory requirements (47). With the increasing incidence of cancer and the lag in drug approvals behind those in North America and Europe, there remains a need for global drug development strategies to ensure broad access to novel therapies (48). Despite efforts like the Bridging Study Evaluation in Taiwan to enhance drug development efficiency (49), hurdles attributed to specific local regulatory requirements persist, such as in the Chinese mainland, which mandates 100 to 300 patients for small molecules and biologics, further delaying access (50). Furthermore, although molecularly targeted therapies show dose consistency, cytotoxic agents often require data from Asian populations due to differences in drug metabolism and toxicity tolerance (51). Additionally, genetic diversity affects drug metabolism and should be considered in clinical trial designs. The shift toward precision medicine and targeted therapies emphasizes optimizing regimens for all populations within global trials. Hence, a globalized approach to drug development, integrating Asia-Pacific from the early stages, can help reduce drug lag and improve treatment access. By addressing regulatory barriers and enhancing multiregional trial participation, the region can benefit from faster approvals and broader access to innovative therapies (48). Recent implementation of such advances has been observed in the global phase III KEYNOTE-A18 study for pembrolizumab plus concurrent chemoradiotherapy, which also demonstrated a higher PFS score among the East Asian population. This study led to the approval of pembrolizumab in the region in 2024 (52), hence implicating advances in the approaches toward inclusion in globalized clinical trials.

There are limitations to the current study. Due to the small sample size of respondents, quantitative instrument analyses were exploratory. Gaps in the data may persist as this study was conducted in selected locations. Moreover, recall biases could have resulted as the measurements investigated were physician-reported. Another limitation is the lack of a survey distinguishing *de novo* stage IVb (FIGO 2018) patients from those who progressed to stage IVb when assessing treatment modalities. This distinction could have provided a more comprehensive interpretation of the patient and treatment landscape. Furthermore, as this survey was conducted and completed prior to the introduction of other novel regimens, such as TIVDAK, we were unable to capture antibody–drug conjugate insights.

### Conclusion

This is one of the very few studies reporting the current mCC treatment landscape and challenges faced across five locations in the Asia-Pacific. The study reported approximately 11% of cervical cancer cases as mCC. Most patients were aged 41 to 60 years, experienced hypertension, and had an ECOG status of 1 to 2. Important treatment choice drivers included treatment guidelines, patient characteristics, and drug characteristics, such as affordability, proximity to treatment centers, and drug safety and efficacy. Preferred treatments included ST or radiotherapy + ST, with challenges centered around funding, costs, accessibility, and availability. These findings suggest the need for effective management of mCC and the high cost of treatments through the potential involvement and collaborative efforts of communities, governments, private sectors, and nonprofit organizations. Global or regional frameworks for programs may not necessarily be relevant or practical across all locations, as evidenced by the current findings from the varied healthcare systems. However, key learnings from successes in other geographies prior to adaptation at a local level could prove beneficial. The funding gap for noncommunicable diseases, including cervical cancer, is significant and will continue to grow without intervention. Efforts to address the unmet needs of patients with mCC should focus on improving accessibility to screening and innovative therapies. Health authorities should ensure adequate reimbursement policies that could contribute to reducing the number of late-stage diagnoses, work productivity losses, and treatment costs while increasing optimal patient outcomes.

## Supplementary Material

Table S1 Table S1 shows the different first-line treatment modalities received by mCC patients

Table S2 Table S2 shows the patient profiles respondents considered for biomarker testing

Table S3 Table S3 shows the importance of various factors influencing physicians' treatment choices for mCC patients across different locations

Table S4 Table S4 shows the top three unmet needs influencing treatment options across locations

Table S5 Table S5 shows access-related challenges reported by respondents in locations where bevacizumab is not publicly reimbursed

Table S6 Table S6 shows access-related challenges reported by respondents in locations where bevacizumab is publicly reimbursed

Table S7 Table S7 shows the current (March 2025) reimbursement status of treatment regimens.

Figure S1 Figure S1 provides an overview of the respondent population across study locations

Figure S2 Figure S2 shows the proportion of mCC patients tested for various biomarkers

Figure S3 Figure S3 shows respondents' likelihood of testing their patients for biomarkers in the future

Figure S4 Figure S4 shows respondents' awareness of the public reimbursement status of bevacizumab in their locations
