# Supplementary material for: Metastatic Cervical Cancer in the Asia-Pacific Region: Current Treatment Landscape and Barriers
Source: Cancer Res Commun. 2025 Aug 26;5(8):1429–40. doi: 10.1158/2767-9764.CRC-24-0647 (PMC12378444; doi:10.1158/2767-9764.CRC-24-0647)
Supplement: Table S1 — shows the different first-line treatment modalities received by mCC patients [file crc-24-0647_table_s1_suppst1.docx]

**Table S1.** Treatment modalities received by mCC patients.

|  | **Region (%)** | **By Location** | | | | |
| --- | --- | --- | --- | --- | --- | --- |
|  |  | **CN (%)** | **AU (%)** | **KR (%)** | **PH (%)** | **TW (%)** |
| ST only | 43.6 | 30.9 | 53.9 | 62.8 | 56.2 | 39.7 |
| RT+ST (including CCRT) | 33.4 | 34.2 | 27.8 | 27.0 | 29.2 | 47.5 |
| RT only | 7.4 | 10.7 | 3.4 | 2.7 | 7.8 | 6.4 |
| No therapy/Observation for primary disease (may still receive pain medication, palliative radiation therapy, TCM alone, or other supportive care) | 6.5 | 8.1 | 8.7 | 4.8 | 4.4 | 3.6 |
| Surgical resection + RT + ST | 3.0 | 6.1 | 0.9 | 0.5 | 0.3 | 0.8 |
| Surgical resection + ST | 2.3 | 3.9 | 0.6 | 2.0 | 0.9 | 0.6 |
| Surgical resection + RT | 1.5 | 3.0 | 0.6 | 0.0 | 0.6 | 0.6 |
| Surgical resection only | 0.9 | 1.8 | 0.0 | 0.3 | 0.1 | 0.3 |
| Palliative treatment | 0.6 | 0.0 | 4.1 | 0.0 | 0.0 | 0.0 |
| TCM | 0.3 | 0.6 | 0.0 | 0.0 | 0.0 | 0.0 |
| Targeted Therapy | 0.2 | 0.3 | 0.0 | 0.0 | 0.3 | 0.0 |
| Immunotherapy | 0.1 | 0.2 | 0.0 | 0.0 | 0.3 | 0.0 |
| Opt for alternative medicine and herbal | 0.1 | 0.0 | 0.0 | 0.0 | 0.0 | 0.6 |
| Clinical trials | 0.1 | 0.2 | 0.0 | 0.0 | 0.0 | 0.0 |
| Family support and opinion | 0.0 | 0.1 | 0.0 | 0.0 | 0.0 | 0.0 |
| Financial capability | 0.0 | 0.1 | 0.0 | 0.0 | 0.0 | 0.0 |

*AU, Australia; CCRT, concurrent chemoradiotherapy; CN, Chinese Mainland; KR, South Korea; PH, Philippines; RT, radiotherapy; ST, systemic therapy; TCM, Traditional Chinese medicine, TW, Taiwan.*
