# Supplementary material for: Metastatic Cervical Cancer in the Asia-Pacific Region: Current Treatment Landscape and Barriers
Source: Cancer Res Commun. 2025 Aug 26;5(8):1429–40. doi: 10.1158/2767-9764.CRC-24-0647 (PMC12378444; doi:10.1158/2767-9764.CRC-24-0647)
Supplement: Table S2 — shows the patient profiles respondents considered for biomarker testing [file crc-24-0647_table_s2_suppst2.docx]

**Table S2.** Patient profiles considered for biomarker testing.

| **PD-L1 Expression** | **Biomarker Status (e.g., MSI/MMR, TMB, etc.)** |
| --- | --- |
| **CN and KR:**  Will try to provide to all metastatic / advanced patients, if possible; however, patient affordability matters Patients actively looking for treatment options (e.g., immunotherapy) and are still fit for treatments | **CN and KR:**   Will try to provide to all metastatic / advanced patients, if possible; but patient affordability matters Patients with family history of cancer (CN) Patients actively looking for treatment options (e.g., immunotherapy) Patients with less common tumor type |
| **TW:** Younger patients Patients with good financial stability Metastatic patients who have failed first line of therapy | **TW:** Younger patients Patients with good financial stability Metastatic patients who have failed first line of therapy |
| *"Patients who are confirmed to have advanced or metastatic cervical cancer.” – CS00024, KR* | *“Targeted therapy or immuno-oncology would be the trend in the future, so I think to perform it on all patients at stage IVb in order to accumulate some data in selecting drugs. Its use is currently being considered in patients who are financially well off, want treatment, and don’t care much about the treatment cost.” – CS00017, KR* |

*AU, Australia; CN, Chinese Mainland; KR, South Korea; MSI/MMR, microsatellite instability/mismatched repair; PD-L1, programmed death-ligand 1; PH, Philippines; TMB, tumor mutational burden; TW, Taiwan.*
