# Supplementary material for: Metastatic Cervical Cancer in the Asia-Pacific Region: Current Treatment Landscape and Barriers
Source: Cancer Res Commun. 2025 Aug 26;5(8):1429–40. doi: 10.1158/2767-9764.CRC-24-0647 (PMC12378444; doi:10.1158/2767-9764.CRC-24-0647)
Supplement: Table S3 — shows the importance of various factors influencing physicians' treatment choices for mCC patients across different locations [file crc-24-0647_table_s3_suppst3.docx]

**Table S3.** Location-specific responses on factors influencing physicians' treatment choices for mCC patients. The importance of each factors was rated on a score of 1 (not important at all) to 10 (extremely important), with scores categorized into the top 3 boxes (10/9/8), middle 4 boxes (7/6/5/4), and bottom 3 boxes (3/2/1).

| **Level of importance** | **CN (%)** | | | **AU (%)** | | | **KR (%)** | | | **PH (%)** | | | **TW (%)** | | |
| --- | --- | --- | --- | --- | --- | --- | --- | --- | --- | --- | --- | --- | --- | --- | --- |
|  | T3B | Mid4 | B3B | T3B | Mid4 | B3B | T3B | Mid4 | B3B | T3B | Mid4 | B3B | T3B | Mid4 | B3B |
| **Treatment guidelines** | | | | | | | | | | | | | | | |
| NCCN guidelines | 81.8 | 18.2 | 0.0 | 47.1 | 47.1 | 5.9 | 95.0 | 5.0 | 0.0 | 88.2 | 5.9 | 5.9 | 100.0 | 0.0 | 0.0 |
| Payor/Provider reimbursement guidelines/criteria | 72.7 | 25.5 | 1.8 | 17.7 | 41.2 | 41.2 | 85.0 | 15.0 | 0.0 | 52.9 | 29.4 | 17.7 | 88.9 | 11.1 | 0.0 |
| Hospital guidelines | 38.2 | 49.1 | 12.7 | 29.4 | 64.7 | 5.9 | 45.0 | 25.0 | 30.0 | 76.5 | 17.7 | 5.9 | 88.9 | 11.1 | 0.0 |
| ESMO guidelines | 0.0 | 0.0 | 0.0 | 52.9 | 35.3 | 11.8 | 0.0 | 0.0 | 0.0 | 0.0 | 0.0 | 0.0 | 61.1 | 38.9 | 0.0 |
| KSGO/KGOG guidelines | 0.0 | 0.0 | 0.0 | 0.0 | 0.0 | 0.0 | 80.0 | 10.0 | 10.0 | 0.0 | 0.0 | 0.0 | 0.0 | 0.0 | 0.0 |
| NHRI guidelines | 0.0 | 0.0 | 0.0 | 0.0 | 0.0 | 0.0 | 0.0 | 0.0 | 0.0 | 0.0 | 0.0 | 0.0 | 50.0 | 44.4 | 5.6 |
| SGOP clinical guidelines | 0.0 | 0.0 | 0.0 | 0.0 | 0.0 | 0.0 | 0.0 | 0.0 | 0.0 | 94.1 | 0.0 | 5.9 | 0.0 | 0.0 | 0.0 |
| **Patient characteristics** | | | | | | | | | | | | | | | |
| Current disease stage | 80.0 | 20.0 | 0.0 | 88.2 | 11.8 | 0.0 | 90.0 | 10.0 | 0.0 | 100.0 | 0.0 | 0.0 | 94.4 | 5.6 | 0.0 |
| ECOG status | 80.0 | 18.2 | 1.8 | 94.1 | 5.9 | 0.0 | 85.0 | 15.0 | 0.0 | 94.1 | 5.9 | 0.0 | 72.2 | 27.8 | 0.0 |
| Comorbidities | 60.0 | 40.0 | 0.0 | 52.9 | 47.1 | 0.0 | 50.0 | 45.0 | 5.0 | 76.5 | 23.5 | 0.0 | 55.6 | 44.4 | 0.0 |
| Affordability | 49.1 | 49.1 | 1.8 | 35.3 | 17.7 | 47.1 | 55.0 | 35.0 | 10.0 | 82.4 | 17.7 | 0.0 | 72.2 | 27.8 | 0.0 |
| PD-L1 status | 49.1 | 47.3 | 3.6 | 23.5 | 76.5 | 0.0 | 55.0 | 45.0 | 0.0 | 29.4 | 47.1 | 23.5 | 66.7 | 33.3 | 0.0 |
| Patient's age | 32.7 | 61.8 | 5.5 | 35.3 | 64.7 | 0.0 | 65.0 | 35.0 | 0.0 | 70.6 | 29.4 | 0.0 | 33.3 | 66.7 | 0.0 |
| Biomarker status | 36.4 | 60.0 | 3.6 | 29.4 | 47.1 | 23.5 | 55.0 | 40.0 | 5.0 | 47.1 | 41.2 | 11.8 | 50.0 | 50.0 | 0.0 |
| Proximity to treatment center (e.g., RT) | 21.8 | 56.4 | 21.8 | 35.3 | 52.9 | 11.8 | 25.0 | 50.0 | 25.0 | 88.2 | 11.8 | 0.0 | 44.4 | 44.4 | 11.1 |
| Patient risk factors (e.g., previous pregnancies, smoking) | 29.1 | 50.9 | 20.0 | 23.5 | 47.1 | 29.4 | 40.0 | 45.0 | 15.0 | 64.7 | 29.4 | 5.9 | 22.2 | 66.7 | 11.1 |
| Patient's knowledge about the disease | 21.8 | 65.5 | 12.7 | 23.5 | 70.6 | 5.9 | 25.0 | 65.0 | 10.0 | 70.6 | 29.4 | 0.0 | 16.7 | 83.3 | 0.0 |
| Patient preference | 12.7 | 61.8 | 25.5 | 35.3 | 58.8 | 5.9 | 10.0 | 75.0 | 15.0 | 88.2 | 11.8 | 0.0 | 22.2 | 72.2 | 5.6 |
| Fertility preservation | 7.3 | 40.0 | 52.7 | 5.9 | 35.3 | 58.8 | 20.0 | 45.0 | 35.0 | 5.9 | 64.7 | 29.4 | 11.1 | 50.0 | 38.9 |
| **Drug characteristics** | | | | | | | | | | | | | | | |
| Efficacy (ORR, OS, PFS) | 81.8 | 18.2 | 0.0 | 88.2 | 11.8 | 0.0 | 90.0 | 10.0 | 0.0 | 94.1 | 5.9 | 0.0 | 100.0 | 0.0 | 0.0 |
| Safety (SE, tolerability) | 78.2 | 21.8 | 0.0 | 76.5 | 23.5 | 0.0 | 85.0 | 15.0 | 0.0 | 94.1 | 5.9 | 0.0 | 100.0 | 0.0 | 0.0 |
| Accessibility (availability in hospital formulary) | 54.6 | 41.8 | 3.6 | 58.8 | 17.7 | 23.5 | 75.0 | 20.0 | 5.0 | 82.4 | 17.7 | 0.0 | 88.9 | 5.6 | 5.6 |
| Reimbursed/subsidized/under insurance scheme/self-pay | 60.0 | 40.0 | 0.0 | 41.2 | 29.4 | 29.4 | 80.0 | 20.0 | 0.0 | 64.7 | 29.4 | 5.9 | 94.4 | 5.6 | 0.0 |
| Treatment cost | 52.7 | 47.3 | 0.0 | 41.2 | 23.5 | 35.3 | 60.0 | 40.0 | 0.0 | 94.1 | 5.9 | 0.0 | 83.3 | 16.7 | 0.0 |
| Treatment duration | 23.6 | 72.7 | 3.6 | 11.8 | 70.6 | 17.7 | 40.0 | 55.0 | 5.0 | 64.7 | 35.3 | 0.0 | 55.6 | 38.9 | 5.6 |
| Administration (oral, injection) | 25.5 | 67.3 | 7.3 | 17.7 | 52.9 | 29.4 | 35.0 | 55.0 | 10.0 | 64.7 | 35.3 | 0.0 | 33.3 | 66.7 | 0.0 |
|  |  |  |  |  |  |  |  |  |  |  |  |  |  |  |  |

*AU, Australia; B3B, bottom 3 boxes; CN, Chinese Mainland; ECOG, European Cooperation Oncology Group; ESMO, European Society of Medical Oncology; KR, South Korea; KSGO/KGOG, Korean Society of Gynecologic Oncology/Korean Gynecologic Oncology Group; NCCN, National Comprehensive Cancer Network; Mid4, middle 4 boxes, NHRI, National Health Research Institute; ORR, overall response rate; OS, overall survival; PD-L1, programmed death-ligand 1; PFS, progression-free survival; PH, Philippines; SE, serious events; RT, radiotherapy; SGOP, Society of Gynecologic Oncologists of the Philippines; T3B, top 3 boxes; TW, Taiwan.*
