# Supplementary material for: Metastatic Cervical Cancer in the Asia-Pacific Region: Current Treatment Landscape and Barriers
Source: Cancer Res Commun. 2025 Aug 26;5(8):1429–40. doi: 10.1158/2767-9764.CRC-24-0647 (PMC12378444; doi:10.1158/2767-9764.CRC-24-0647)
Supplement: Table S4 — shows the top three unmet needs influencing treatment options across locations [file crc-24-0647_table_s4_suppst4.docx]

**Table S4**. Location-specific responses on top three unmet needs impacting treatment options. Unmet needs were scored using a ranking system based on the level of impact: 1 = biggest impact, 2 = second biggest impact, 3 = third biggest impact.

|  | **CN (%)** | | |  | **AU (%)** | | |  | **KR (%)** | | |  | **PH (%)** | | |  | **TW (%)** | | |
| --- | --- | --- | --- | --- | --- | --- | --- | --- | --- | --- | --- | --- | --- | --- | --- | --- | --- | --- | --- |
|  | **RANK** | | |  | **RANK** | | |  | **RANK** | | |  | **RANK** | | |  | **RANK** | | |
|  | **1** | **2** | **3** |  | **1** | **2** | **3** |  | **1** | **2** | **3** |  | **1** | **2** | **3** |  | **1** | **2** | **3** |
| Poor patient prognosis with current treatment options | 29.1 | 21.8 | 9.1 |  | 29.4 | 17.7 | 17.7 |  | 15.0 | 10.0 | 25.0 |  | 29.4 | 23.5 | 5.9 |  | 27.8 | 16.7 | 11.1 |
| Patient affordability | 14.6 | 18.2 | 23.6 |  | 0.0 | 5.9 | 5.9 |  | 30.0 | 10.0 | 25.0 |  | 52.9 | 5.9 | 11.8 |  | 22.2 | 22.2 | 27.8 |
| Limited treatment options available | 21.8 | 16.4 | 14.6 |  | 17.7 | 23.5 | 11.8 |  | 25.0 | 25.0 | 15.0 |  | 5.9 | 11.8 | 17.7 |  | 22.2 | 33.3 | 11.1 |
| Unavailability of drugs in hospital formulary | 7.3 | 5.5 | 5.5 |  | 29.4 | 23.5 | 5.9 |  | 0.0 | 5.0 | 0.0 |  | 0.0 | 23.5 | 23.5 |  | 0.0 | 0.0 | 0.0 |
| Barriers towards access to Bevacizumab | 3.6 | 1.8 | 7.3 |  | 5.9 | 5.9 | 5.9 |  | 20.0 | 10.0 | 5.0 |  | 5.9 | 23.5 | 11.8 |  | 0.0 | 0.0 | 0.0 |
| National healthcare policy / disease prioritisation | 3.6 | 16.4 | 7.3 |  | 5.9 | 0.0 | 11.8 |  | 0.0 | 15.0 | 5.0 |  | 0.0 | 5.9 | 0.0 |  | 16.7 | 16.7 | 22.2 |
| Adequate healthcare infrastructure (e.g., availability of adequate RT equipment, etc.) | 7.3 | 3.6 | 1.8 |  | 0.0 | 5.9 | 0.0 |  | 0.0 | 10.0 | 10.0 |  | 0.0 | 0.0 | 5.9 |  | 5.6 | 0.0 | 11.1 |
| COVID implications | 5.5 | 3.6 | 10.9 |  | 5.9 | 5.9 | 17.7 |  | 0.0 | 0.0 | 0.0 |  | 0.0 | 0.0 | 0.0 |  | 0.0 | 5.6 | 5.6 |
| Uneven distribution of medical resources | 1.8 | 1.8 | 14.6 |  | 0.0 | 5.9 | 5.9 |  | 0.0 | 5.0 | 5.0 |  | 5.9 | 0.0 | 11.8 |  | 5.6 | 5.6 | 5.6 |
| Limited referral to cancer centers | 1.8 | 5.5 | 3.6 |  | 5.9 | 5.9 | 11.8 |  | 0.0 | 0.0 | 5.0 |  | 0.0 | 5.9 | 5.9 |  | 0.0 | 0.0 | 0.0 |
| Others^*^ | 3.6 | 5.5 | 1.8 |  | 0.0 | 0.0 | 5.9 |  | 10.0 | 10.0 | 5.0 |  | 0.0 | 0.0 | 5.9 |  | 0.0 | 0.0 | 5.6 |

**^*^**Lack of skilled medical personnel, nutrition, opt for alternative medicine and herbal, patients’ willingness to receive treatment, profitability of the hospital is poor, immuno-oncology's reimbursement is not applied/Limitation in the reimbursement criteria, give up treatment due to old age, side effects due to systemic therapy, and not applicable.

*AU, Australia; CN, Chinese mainland; COVID, Coronavirus disease; KR, South Korea; PH, Philippines; RT, radiotherapy; TW, Taiwan.*
