# Supplementary material for: Metastatic Cervical Cancer in the Asia-Pacific Region: Current Treatment Landscape and Barriers
Source: Cancer Res Commun. 2025 Aug 26;5(8):1429–40. doi: 10.1158/2767-9764.CRC-24-0647 (PMC12378444; doi:10.1158/2767-9764.CRC-24-0647)
Supplement: Table S5 — shows access-related challenges reported by respondents in locations where bevacizumab is not publicly reimbursed [file crc-24-0647_table_s5_suppst5.docx]

**Table S5.** Access-related challenges when bevacizumab is not publicly reimbursed.

| **Access issues** | **Verbatims** |
| --- | --- |
| **Financial barriers**  Not all patients can afford bevacizumab  Some patients with private insurance can afford | *"Patient mostly need to be supported financially whether public or private insurance" - CS50017, PH*  *"They can gain access but not all can afford. Not all self-pay can afford because of the cost." - CS50006, PH* |
| **Concerns over treatment efficacy and side effects**  Uncertain about whether bevacizumab is effective for patients  Some patients cannot tolerate the side effects/adverse events from the treatment (e.g., patients with poor gastrointestinal conditions) | *“The challenges are expectations on treatment efficacy and if there’s complication using this drug.” – CS100023, CN*  *"To use bevacizumab on patients after RT, patients might worry about occurrence of fistula." - CS100015, CN* |
| **Direct and indirect costs of coming to treatment centers**  Not all hospitals have bevacizumab, causing access problems for patients | *“Delivery issues, it’s like ordering and delivery services, they have problem with those.”– CS50012, PH*  *“No, it’s expensive, no available med representative. I have one patient that is going to Manila just to buy” – CS50012, PH* |
| **Issues with patient tolerability and treatment suitability**  Some patients are unsuitable for bevacizumab or cannot tolerate the treatment due to their comorbid and physical conditions | *“Some patients are not considered as ideal candidates for bevacizumab.” – CS100017, CN*  *“It cannot be used on patients with contraindications.” – CS100025, CN* |

*CN, Chinese Mainland; PH, Philippines; RT, radiotherapy.*
