# Supplementary material for: Metastatic Cervical Cancer in the Asia-Pacific Region: Current Treatment Landscape and Barriers
Source: Cancer Res Commun. 2025 Aug 26;5(8):1429–40. doi: 10.1158/2767-9764.CRC-24-0647 (PMC12378444; doi:10.1158/2767-9764.CRC-24-0647)
Supplement: Table S6 — shows access-related challenges reported by respondents in locations where bevacizumab is publicly reimbursed [file crc-24-0647_table_s6_suppst6.docx]

**Table S6.** Access-related challenges when bevacizumab is publicly reimbursed.

| **Access issues** | **Verbatims** |
| --- | --- |
| **Issues with patient tolerability and treatment suitability**  Metastatic patients generally have poor prognosis and might be less tolerable towards bevacizumab if they have other comorbid conditions such as hypertension, etc. | *“If they have bowel diseases like bowel involvement, we don't give it; or they have comorbidity like hypertension, or history of deep vein thrombosis, or high blood pressure.” – CS00105, AU* |
| **Direct and indirect costs of coming to treatment centers**  Bevacizumab might not be readily available in some hospitals, especially smaller units  Patients need to travel to hospitals that have bevacizumab but further away from their home | *“It's not readily available in certain smaller units.” – CS00117, AU* |
| **Concerns over treatment efficacy and side effects**  Treatment efficacy might not be as good as expected  Some patients cannot tolerate the side effects/adverse events from the treatment | *“Efficacy is not as good as expected.” – CS200003, TW* |
| **Lack of medical resources**  Lack of medical resources and equipment such as hospital beds for patients receiving the treatment | *“Basically, it goes around chemo beds for these patients. It’s given by medical oncologist. They only have a certain number of chairs/beds, they can only give treatment to such number of patients per day. If there’s a backlog, then patients cannot be fitted in on a timely matter. So, it’s about the resources of chemo department.” – CS00106, AU* |

*AU, Australia; TW, Taiwan*
