# Supplementary material for: Metastatic Cervical Cancer in the Asia-Pacific Region: Current Treatment Landscape and Barriers
Source: Cancer Res Commun. 2025 Aug 26;5(8):1429–40. doi: 10.1158/2767-9764.CRC-24-0647 (PMC12378444; doi:10.1158/2767-9764.CRC-24-0647)
Supplement: Table S7 — shows the current (March 2025) reimbursement status of treatment regimens. [file crc-24-0647_table_s7_suppst7.docx]

**Table S7. Current (March 2025) reimbursement status of treatment regimens.**

|  | **CN** | **AU** | **KR** | **PH** | **TW** | **References**^*^ |
| --- | --- | --- | --- | --- | --- | --- |
| Chemotherapy | Yes | Yes | Yes  (With co-payment) | No | Yes | [1, 2, 4, 5, 6] |
| Radiotherapy | Yes | Yes | Yes  (With co-payment) | No | Yes | [3, 5, 7] |
| Bevacizumab | Yes | Yes | Yes | No | Yes | [1, 2, 4, 6] |
| *Pembrolizumab* | *No* | *Yes* | *No* | *No* | *No* | [2] |

Chemotherapy: Reflective of chemotherapy commonly used for mCC patients such as cisplatin, carboplatin, paclitaxel

*Abbreviations: AU, Australia; CN, Chinese Mainland; KR, South Korea; PH, Philippines; TW, Taiwan.*

**^*^**References:

1. Reimbursement Drug List Search – China Health Insurance Website - China Health Insurance Website [Internet]. [cited 2025 May 14]. Available from: <https://www.zgylbx.com/index.php?m=content&c=index&a=lists&catid=105>
2. 4169-Cervical recurrent or metastatic cARBOplatin, PACLitaxel, beVACizumab and pembrolizumab | eviQ [Internet]. [cited 2025 May 14]. Available from: https://www.eviq.org.au/medical-oncology/gynaecological/cervical/4169-cervical-recurrent-or-metastatic-carboplatin
3. NSW Government. Gynaecological | eviQ [Internet]. [cited 2025 May 15]. Available from: https://www.eviq.org.au/radiation-oncology/gynaecological
4. FAQ < Drugs and Therapy for Cancer Diseases < Drug Standards Information < System · Policy < Health Insurance Review and Assessment Service [Internet]. [cited 2025 May 14]. Available from: <https://www.hira.or.kr/bbsDummy.do?brdBltNo=45619&brdScnBltNo=4&pgmid=HIRAA030023080000>
5. BMPlus. Cervical cancer coverage: A top priority of PhilHealth in Universal Health Care | BMPlus [Internet]. BusinessMirror. 2023 [cited 2025 May 14]. Available from: <https://businessmirror.com.ph/2023/06/09/cervical-cancer-coverage-a-top-priority-of-philhealth-in-universal-health-care/>
6. National Health Insurance Administration, Ministry of Health and Welfare. 9.37. Bevacizumab(e.g., Avastin)_ Full Text of Revised Reimbursement Criteria [Internet]. Available from: <https://info.nhi.gov.tw/api/INAE3000/INAE3000S01/getPDF?DurgFileName=9.37._20250401.pdf>
7. cancer.commonhealth.com.tw. The latest treatment for cervical cancer is immunotherapy! Learn about treatment costs and post-surgery side effects [Internet]. cancer.commonhealth.com.tw. 2022 [cited 2025 May 14]. Available from: <https://cancer.commonhealth.com.tw/article/334>
