# Supplementary figures and images for: Metastatic Cervical Cancer in the Asia-Pacific Region: Current Treatment Landscape and Barriers
Source: Cancer Res Commun. 2025 Aug 26;5(8):1429–40. doi: 10.1158/2767-9764.CRC-24-0647 (PMC12378444; doi:10.1158/2767-9764.CRC-24-0647)

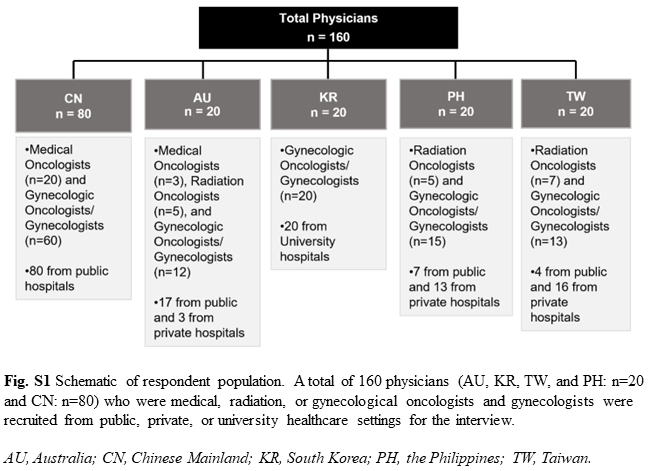

Supplement: Figure S1 — provides an overview of the respondent population across study locations [file crc-24-0647_figure_s1_suppsf1.png]

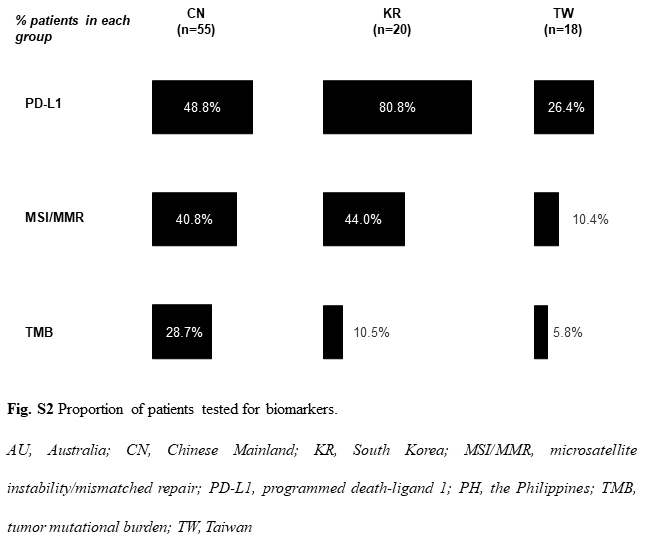

Supplement: Figure S2 — shows the proportion of mCC patients tested for various biomarkers [file crc-24-0647_figure_s2_suppsf2.png]

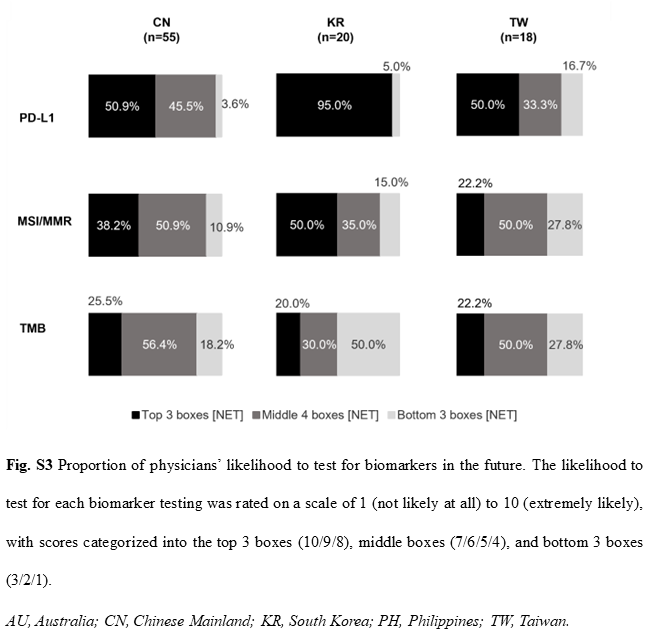

Supplement: Figure S3 — shows respondents' likelihood of testing their patients for biomarkers in the future [file crc-24-0647_figure_s3_suppsf3.png]

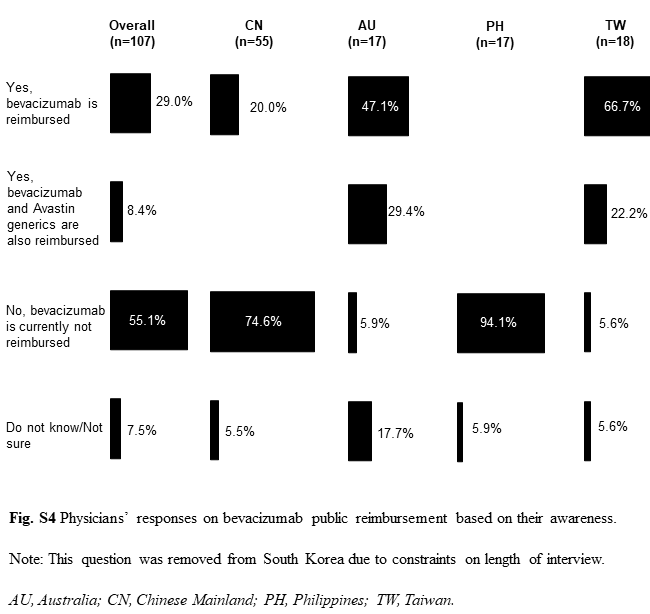

Supplement: Figure S4 — shows respondents' awareness of the public reimbursement status of bevacizumab in their locations [file crc-24-0647_figure_s4_suppsf4.png]
